# Rehabilitation for the management of knee osteoarthritis using comprehensive traditional Chinese medicine in community health centers: study protocol for a randomized controlled trial

**DOI:** 10.1186/1745-6215-14-367

**Published:** 2013-11-04

**Authors:** Hu Yan, Youxin Su, Lidian Chen, Guohua Zheng, Xueyi Lin, Baojun Chen, Bihong Zhou, Qing Zhang

**Affiliations:** 1 Fujian University of Traditional Chinese Medicine, 1 Huatuo St, Shangjie, Minhou, Fuzhou, Fujian, China

**Keywords:** Knee osteoarthritis, Traditional Chinese medicine, Rehabilitation, Clinical trial

## Abstract

**Background:**

It is becoming increasingly necessary for community health centers to make rehabilitation services available to patients with osteoarthritis of the knee. However, for a number of reasons, including a lack of expertise, the small size of community health centers and the availability of only simple medical equipment, conventional rehabilitation therapy has not been widely used in China. Consequently, most patients with knee osteoarthritis seek treatment in high-grade hospitals. However, many patients cannot manage the techniques that they were taught in the hospital. Methods such as acupuncture, *tuina*, Chinese medical herb fumigation-washing and t’ai chi are easy to do and have been reported to have curative effects in those with knee osteoarthritis. To date, there have been no randomized controlled trials validating comprehensive traditional Chinese medicine for the rehabilitation of knee osteoarthritis in a community health center. Furthermore, there is no standard rehabilitation protocol using traditional Chinese medicine for knee osteoarthritis. The aim of the current study is to develop a comprehensive rehabilitation protocol using traditional Chinese medicine for the management of knee osteoarthritis in a community health center.

**Method/design:**

This will be a randomized controlled clinical trial with blinded assessment. There will be a 4-week intervention utilizing rehabilitation protocols from traditional Chinese medicine and conventional therapy. Follow-up will be conducted for a period of 12 weeks. A total of 722 participants with knee osteoarthritis will be recruited. Participants will be randomly divided into two groups: experimental and control. Primary outcomes will include range of motion, girth measurement, the visual analogue scale, and results from the manual muscle, six-minute walking and stair-climbing tests. Secondary outcomes will include average daily consumption of pain medication, ability to perform daily tasks and health-related quality-of-life assessments. Other outcomes will include rate of adverse events and economic effects. Relative cost-effectiveness will be determined from health service usage and outcome data.

**Discussion:**

The primary aim of this trial is to develop a standard protocol for traditional Chinese medicine, which can be adopted by community health centers in China and worldwide, for the rehabilitation of patients with knee osteoarthritis.

**Trial registration:**

Clinical Trials Registration: ChiCTR-TRC-12002538

## Background

Knee osteoarthritis (KOA) increases in prevalence with age and causes pain, loss of functional independence and psychological impairment, and reduces the overall quality of life of affected individuals [1-3]. Based on epidemiological results, the incidence rate of osteoarthritis in the Chinese population is approximately 10% and the number of patients with KOA is greater than 50 million [4]. KOA has been identified as one of the most serious diseases that can affect an individual during his or her lifetime. It is a degenerative disorder caused by the interplay of genetic, metabolic, biochemical and biomechanical factors and results in pain, swelling and malformation of the knee joint [5]. KOA is prevalent and chronic and is associated with physical and psychological dysfunction. It reduces quality of life by inhibiting or preventing sufferers from carrying out activities such as walking, climbing stairs, doing housework, socializing and engaging in outdoor pursuits [6]. Furthermore, dysfunction due to KOA strongly influences employment status, productivity [7,8] and increases obesity rates [9], especially among older people. Pain, swelling, malformation and muscle weakness are often interrelated, leading to a downward cascade in physical as well as mental function [10-12].

During rehabilitation, patients with KOA require good pain control with few adverse events (AEs). Clinical guidelines advocate conservative non-drug strategies for the treatment of KOA [13], such as muscle-strengthening exercises, electromagnetic therapy and acupuncture. These conventional rehabilitation therapies are focused on controlling pain as well as reducing physical disability and impairment, while minimizing the potentially harmful side effects of medication [14]. These therapies can improve KOA patient outcomes [15], but are often only offered in high-grade hospitals and not in community health centers (CHCs). Consequently, the direct and indirect health-care costs associated with KOA frequently become a major burden to patients. After their discharge, patients will often abandon the rehabilitation therapy techniques they have learned and the KOA will become progressively worse. Therefore, as the patients aging and comorbidities increasing, a more convenient, effective, simple and inexpensive approach to rehabilitation is necessary for those with KOA.

The use of traditional Chinese medicine (TCM) for the treatment of KOA has increased substantially. In Chinese medicine, KOA is considered to originate in the tendons and bones, but manifests as viscera insufficiency and *biao* or *branch* excess. The invasion of evil pathogens, due to wind-cold-dampness, is linked to blood stasis, which then leads to *wei zheng* and *bi zheng*[16]. *Wei zheng* is treated by viscera reinforcement and *bi zheng* is treated by wind-cold-dampness purging and blood activation.

TCM has been used for health care in China for thousands of years. Some TCM rehabilitation methods, such as Chinese medical herbal patches, traditional exercises (developed from simplified t’ai chi and *yi jinjing*) and acupuncture have been reported to improve KOA in patients clinically [17-20]. The comprehensive TCM rehabilitation protocol (CTCMRP) comprises Chinese medical herb fumigation-washing (done to dispel wind-cold-dampness and to activate blood circulation in the knee), traditional exercise training, acupuncture and bloodletting via puncture. CTCMRP can enhance muscle strength, improve balance, reduce knee pain, stimulate the governor vessel and spine, and can regulate the function of the viscera, *qi* and blood in the *zang*-*fu* organs.

However, results from randomized controlled trials examining TCM treatments for KOA are contradictory and there is no standard rehabilitation protocol for KOA treatment using TCM (SRPTCM) [21]. Therefore, in the current study a protocol was designed in which KOA patients will be treated using CTCMRP in CHCs. The data from this study will shed light on the use of TCM for KOA rehabilitation.

## Methods/design

### Study design

**Figure 1:**
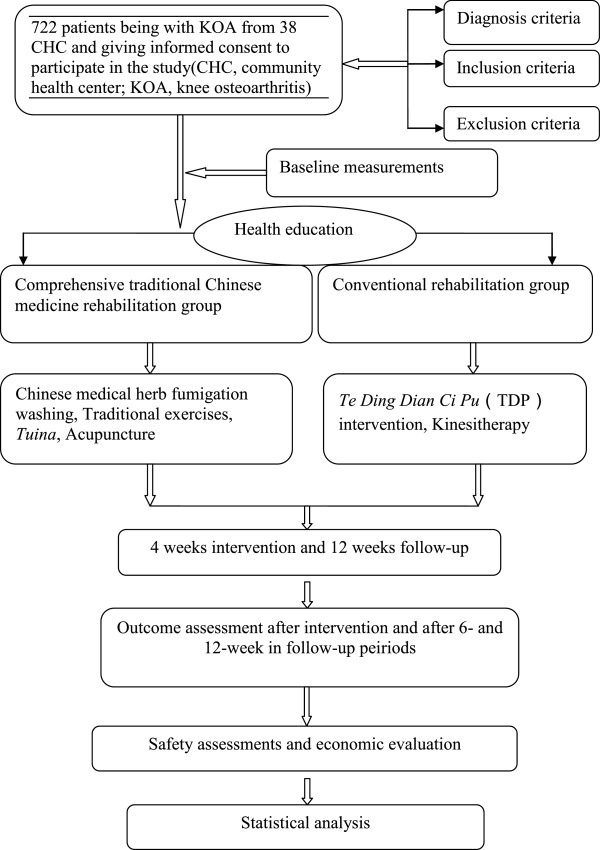
Study flow chart.

This will be a cluster randomized controlled trial with blinded assessment (Figure 1). The trial will comprise a 4-week intervention, using the CTCMRP and conventional therapy (CT), and a 12-week follow-up period. The protocol will be conducted at CHCs run by the Affiliated People’s Hospital of Fujian University of Traditional Chinese Medicine (Fujian province, China), the Comprehensive Traditional and Western Medicine Hospital of Guangdong province (Guangdong province, China), the Chengdu Military Region General Hospital of the Chinese People’s Liberation Army (Sichuan province, China) and the First Affiliated Hospital of Guangzhou University of Traditional Chinese Medicine (Guangdong province, China). To include a broad range of patients, this protocol includes CHCs in different areas. Fujian University of Traditional Chinese Medicine (FJTCM) is responsible for training and supervising rehabilitation therapists at all clinical sites. The evidence-based medicine center, which will be used for documentation and data analysis, is an independent site that performs data monitoring, randomization and statistical evaluation and will be blind to the intervention. Patients will be recruited through CHC outpatient clinics. The Human Research Ethics Committee at Fujian University of Traditional Chinese Medicine (Fujian province, China) approved the study.

### Participants

Patients suffering from pain, swelling, muscle weakness and stiffness of the knee will tested for KOA. All participants will be required to provide signed, informed consent prior to beginning baseline assessment and rehabilitation.

### Inclusion criteria

•Participants will have been diagnosed with KOA according to the criteria in the *Guide Diagnosis of Bone Arthritis* (2007) published by the Chinese Medical Association [22].

•Participants may be male or female and must be between 40 and 75 years of age.

•Participants will be considered to have early- to middle-stage KOA.

•Participants will understand the research and will have signed the informed consent.

•Symptoms will include pain, swelling, stiffness or malfunction of the knee according to the assessment scale.

### Exclusion criteria

Patients are excluded if they have:

•severe knee swelling

•a muscle strength of grade 2 or below according to a manual muscle test

•a history of trauma to the knees

•serious heart, kidney, liver or nervous system disease, or lower limb gangrene due to diabetes.

•lower limb dysfunction caused by spinal, hip or ankle joint disease, or prior knee joint surgery

•a tumor of the knee joint

•a cognitive or psychological disorder

•received traditional exercise training and/or *tuina* for the knee within the last 4 weeks

### Recruitment

Outpatients with KOA will be recruited from participating CHCs in China. All participants will be selected according to the above inclusion and exclusion criteria. Baseline measurements will include the general and demographic data for the subjects, assessment of the knee prior to testing, medication use, previous treatments, KOA complications and duration of KOA symptoms. Due to ethical considerations, non-steroidal, anti-inflammatory drugs will be permitted (and registered) as required for patients with visual analogue scale (VAS) scores over 5. All patients will be asked to refrain from seeking other forms of treatment during the trial. During the follow-up period, patients will be asked to report whether they have received other forms of treatment.

### Randomization and allocation

Following an epidemiological survey, the CHC randomization list for this study will be computer generated using SAS software (Version 8.2, SAS Institute, Cary, NC, USA) and concealed at an independent evidence-based medical center. Employees at the medical center will also be blinded to the intervention. Treatment allocation will start when it has been determined that a study participant meets the inclusion criteria and has signed the informed consent.

The CHC randomization program will assign subjects to each group and each participant’s group number will be displayed (CTCMRP versus CT rehabilitation) on the back of the form. Baseline measurements will not be viewed until the end of the study to ensure blinded assessment. The allocation list will be protected and held by FJTCM. In the event of an emergency, the individual’s randomization code and group allocation can be identified. Therapists at each participating CHC will provide treatment, as usual, to patients who are not eligible or willing to participate.

### Sample size

In cluster randomized trials, the inspection efficiency comes from the random unit number (cluster number) rather than the size. There are two important factors when estimating the sample number: (1) The number within each cluster group and (2) the intraclass correlation coefficient estimate. The total number of subjects (*n*) from all of the CHCs was calculated according to the estimation formula [23]:

$$n=\frac{{\left(\mu_{\alpha }+\mu_{\beta }\right)}^{2}\left[\pi_{1}\left(1-\pi_{1}\right)+\pi_{2}\left(1-\pi_{2}\right)\right]\left[1+\left(m-1\right)\rho \right]}{{\left(\pi_{1}-\pi_{2}\right)}^{2}}$$

In a trial test of this protocol, the curative effect rate after rehabilitation of KOA was 70% in the control group (*π*_1_) and 82.3% in the experimental group (*π*_2_); this will be used as the main index. The number of qualified participants recruited from each CHC (*m*) will not be less than 17. Prior data indicate that the intra-CHC correlation coefficient (*ρ*) is likely to be, at most, 0.020, and*μ*_*α*_ = 0.05 and *μ*_*β*_ = 0.10.

There are 38 CHCs. Based on the formula, each group (control and experimental) will comprise 323 participants. A total number of 722 subjects will be included to allow for a dropout rate of 10%.

### Ethical issues

The Ethics Committee of the FJTCM approved this protocol (Approval No. 2012–031). Each participating center will obtain local institutional review board approval. All study participants will sign an informed consent sheet prior to participation.

### Baseline measurements

Baseline descriptive data will be obtained via a questionnaire (Table 1).

**Table 1:** Baseline descriptive data

**Data**	**Content**
General data	Agent of the person who conducted test, test start date, the patient’s address, name of accompanying family members and the patient’s telephone number
Demographic data of subject	Gender, age, nationality, level of education, profession, height and weight
Assessment of knee before test	Visual analogue scale (VAS), knee girth, knee’s range of motion (ROM), manual muscle test (MMT), stair-climbing ability, walking distance, comprehensive effect, average daily amount of non-steroidal anti-inflammatory drugs taken, activities of daily living (ADL), quality of life (QOL), safety assessments and economic evaluation
Other	Complications, medication use, previous treatment and duration of KOA symptoms

### Interventions

The participants in the experimental group will undergo CTCMRP rehabilitation treatment. This consists of Chinese medical herb fumigation-washing, traditional exercises (developed from simplified t’ai chi and *yi jinjing*), acupuncture and *tuina*. Patients in the control group will undergo CT rehabilitation including physical factor therapy and kinesitherapy. The duration of rehabilitation will be 4 weeks, which is a common duration in clinical practice. During the rehabilitation period the therapist will monitor proper performance, exercise intensity and will progress the exercises, if necessary.

### Health education

Two strategies will be used for health education: (1) Educational brochures on KOA, published by FJTCM, will be sent to participants. Information in these brochures will include the concept, epidemiology, etiology, common symptoms, diagnosis, treatments and precautions that should be taken. (2) The rehabilitation therapist will introduce and explain the contents of the educational brochures to all participants in person.

The aim is to help participants learn about and understand KOA and to implement strategies to help alleviate the disease. Moderate activity, aerobic training (swimming), avoidance of long periods of running or jumping, decreasing or avoiding climbing stairs, and reducing weight if necessary, can all help to improve KOA.

### Experimental group

The experimental group will be treated according to the CTCMRP (Table 2).

**Table 2:** TCM rehabilitation based on syndrome using appropriate technology

**Symptom**	**TCM rehabilitation treatment**
Diagnosis of KOA and inclusion and exclusion criteria	Basic rehabilitation: Chinese medical herb fumigation-washing + traditional exercises (developed from simplified t’ai chi and *yi jinjing*)
Swelling (mild to moderate by knee girth)	Basic rehabilitation + bloodletting by puncturing and cupping
Weak muscle strength (over 3 degrees by MMT)	Basic rehabilitation + *tuina*
Pain (over 5 by VAS)	Basic rehabilitation + acupuncture

In all cases, Chinese medical herb fumigation-washing with traditional exercises (to a basic prescription) will be used. If there is significant swelling in the joint, basic rehabilitation plus cupping and bloodletting will be used. When there is a significant decrease in joint strength, basic rehabilitation plus *tuina* will be used. When there is significant joint pain, basic rehabilitation plus acupuncture will be used.

#### Chinese medical herb fumigation-washing

The right or left knee joint will be washed twice daily, for 6 days, with a decoction of *hai tong pi*[24]. The recipe for this is: *Erythrina indica* Lam (15 g), moxa (15 g), *Phryma leptostachya* (15 g), *Lycopodium clavatum* (15 g), radix aconiti (10 g), radix aconiti agrestis (10 g), *Pericarpium zanthoxyli* (10 g), radix angelicae (10 g), radix clematidis (10 g), cassia twig (10 g), safflower carthamus (5 g) and *Ligusticum wallichii* (5 g). Patients will undergo a 6-day course of treatment on four separate occasions (Table 3).

**Table 3:** **Traditional exercise intervention** (**developed from simplified t**’**ai chi and *yi jinjing***)

**Phase**	**Description and progression**
Phase 1: foot and heel jolt	(i) Stand at attention, heel lift up and look forward for 10 seconds
	(ii) Move the heel back down, tap the ground slightly and keep looking forward
Phase 2: rotate knee	(i) Stand at attention, slightly bend knees, put hands on the ipsilateral knees and look forward
	(ii) Circular movement of knee
Phase 3: heel kick	(i) Lie down with leg bent and hands lying naturally at the side of the body
	(ii) Bend at the hip joint and at the knees, extend the back of the ankle for 15 seconds, kick up the heel while moving the toes toward the shin (plantar flexion) and straighten the knee as far as possible. Do this one leg at a time

#### Traditional exercises

Simplified t*’*ai chi and yi jinjing exercises will be performed twice daily with each patient completing one set of 15 repetitions for both knees (Table 3).

#### Cupping and bloodletting intervention

Patients will undergo cupping as well as being punctured for bloodletting. This will be done twice weekly, at points ST 34 and SP 10, using a percusso-punctator. This will not be done in patients with skin ulcers, dermatitis or hemophilia.

#### Tuina

This treatment is based on the theory of meridians and will be done once a day (15 repetitions of 3 to 5 minutes each) as in clinical practice (Table 4).

**Table 4:** ***Tuina *** **intervention**

**Location**	**Description and progression**
Bladder meridians	Thumb and fingers rub the points from BL 37 to BL 56
Stomach meridians	Thumb and fingers rub the points from ST 34 to ST 36
Spleen meridians	Thumb and fingers rub the point from SP 9 to SP 10
Around the knee	Push the muscle and warm using the palm of the hand and the base of the thumb (the thenar eminence) repeatedly

#### Acupuncture

Patients with a pain score over 3, according to the VAS scale, will undergo acupuncture. While the patient is lying down, needles will be inserted into the following acupuncture points: Ex-LE 5, Ex-LE05, Ex-LE 2 and Ashi. The treatment will last 30 minutes and will be carried once per day. Single-use Huatuo needles (0.35 × 40 mm) will be used and will be disposed of into a bedside sharps container upon completion of treatment.

### Control group

The control group will be treated with physical factor rehabilitation and kinesitherapy [22,25].

#### Thermal design power

Participants will be asked to undergo *Te Ding Dian Ci Pu* (TDP, a machine that can release specific frequency of electromagnetic wave to treat disease) for at least 30 minutes per day. The distance between the TDP and the knee must be from 30 cm to 50 cm.

#### Kinesitherapy

Kinesitherapy is designed to strengthen lower limb muscles and incorporates exercises commonly used in clinical practice. It is based on clinical trials showing that such exercise programs can reduce pain and improve function [26]. The aim of kinesitherapy is to strengthen the quadriceps, hamstrings and other muscles around the knee. Participants will do this twice daily under the direction of the therapist (Table 5).

**Table 5:** Kinesitherapy

**Intervention**	**Description**
Passive movement	(i) The patient sits on a bed with the limb hanging over the edge of a bed. A rolled towel or pad is placed under the shallow depression at the back of the knee joint (popliteal fossa).
	(ii) The therapist holds the bottom (distal) of the lower leg and pulls toward the foot for 10 seconds. This is repeated five times consecutively.
	(iii) The therapist uses his or her hands to flex and extend the knee. Patients will be informed that this will be a passive motion and may uncomfortable but should not be painful.
Active movement	(i) The participant lies on his or her back, lifts and straightens one leg with a weight attached to the ankle. The heel is lifted so that it is approximately 18 cm above the bed and held in that position for 30 seconds. This is repeated three times consecutively.
	(ii) The participant lies on his or her back, stretches out the knee and performs a set of 15 quadriceps contractions keeping the leg in the same position. This is repeat twice a day for 4 weeks.

### Timelines

Project funding was approved in July 2012 and commenced in October 2012. Ethics approval was obtained from the Medical Ethics Committee of FJTCM in September 2012. Recruitment commenced in October 2012 and will be completed in October 2013. The trial is due for completion in October 2015 at which time all participants will have completed the 3-month follow-up. Outcomes will be assessed at baseline, and at 4-, 10- and 12-weeks post-baseline. Telephone contact and follow-up clinic visits will be used to assess participant adherence to the study protocol.

### Follow-up

During the 12-week follow-up period, trained personnel will contact the participants each week via telephone. Participants will be asked about the KOA and whether they have received other treatments. Participants will be asked to attend a follow-up clinic at 6 and 12 weeks to assess pain, swelling, range of motion, muscle strength, average walking distance, stair climbing ability, activities of daily living (ADL), quality of life (QOL) and medication usage.

### Outcome assessment

Outcome measures have been selected based on those recommended for clinical trials of KOA [27] (Table 6).

**Table 6:** Summary of measures to be collected

**Outcome measures**	**Data collection instrument**
**Primary outcome measures**	
Pain	Visual analogue scale (VAS)
Swelling	Girth measurement
Knee range of motion	Range of motion (ROM)
Muscle strength	Manual muscle test (MMT)
Walking distance	Six-minute walking test (6 MW)
The time to ascend and descend a flight of stairs	Stair-climbing test (SCT)
**Secondary outcomes**	
Pain	Average daily consumption of drugs
Activities of daily living	Modified Barthel index (MBI)
Quality of life	Medical outcomes short form 36 health questionnaire (SF-36)
**Other outcomes**	
Adverse events	Safety assessments
Cost	Economic evaluation

### Primary outcomes

Average knee pain will be self-assessed using a 100 mm visual analogue scale (VAS) with terminal descriptors of ‘no pain’ and ‘worst pain possible’. The VAS pain measurement has demonstrated reliability in KOA [27].

Knee girth will be determined using a flexible plastic measuring tape to measure the transverse plane circumference of the knee at the mid-patellar height, with the patient in a supine position. Girth measurement has shown acceptable reliability for determination of gross changes in knee swelling after knee surgery [28].

The knee’s range of motion (ROM) will be measured using a standard long-arm goniometer. To determine knee flexion ROM, patients will be asked to slide the heel actively towards the buttocks and maximal active knee flexion will be measured. Knee ROM in patients with KOA has been shown to be adequately reliable with a coefficient of 0.96 for flexion and 0.81 for extension [29].

The manual muscle test (MMT) will be performed according to Medical Research Council (MRC) guidelines [30]. The British MRC has developed a muscle scale that grades muscle strength between 0 and 5 [31]. According to the muscle scale, 0 = no palpable contraction, total paralysis; 1 = flicker or trace of muscle contractions; 2 = active movement with gravity eliminated; 3 = full range with gravity eliminated; 3– = partial range against gravity; 3+ = full range against gravity with no resistance possible; 4 = slight resistance; 4– = mild resistance; 4+ = moderate resistance; 5– = barely detectable weakness and 5+ = normal motor power.

Walking performance will be assessed using the six-minute walking test (6 MW). This measures the distance that participants are able to walk in 6 minutes while wearing their usual footwear. Patients will be allowed to use a cane, if necessary. Standardized feedback will be provided to patients during each test as described previously [32]. The 6 MW test will be done twice and the test results averaged. A longer walking distance than baseline will indicate better walking performance. The 6 MW test has excellent test-retest reliability from 0.95 to 0.97 and a low coefficient of variation (10.4%) [33].

The stair-climbing test (SCT) is a physical performance test that measures the time it takes to ascend and descend a flight of stairs. Patients will be asked to complete the test as quickly as they can while still feeling safe and comfortable. Patients will be allowed to use a device if necessary, though participants will be encouraged to perform the test without the aid of a device if possible. Rejeski [34] found that a similar stair-climbing task had an excellent test–retest reliability coefficient of 0.93.

Based on the *Guidelines for the Clinical Research of Chinese Medicine New Drugs* (2002) [35], the curative effect for KOA is calculated as:

$$\begin{array}{l} \mathrm{Curative}\;\mathrm{effect}\\ \;=\left(\mathrm{Total}\;\mathrm{score}\;\mathrm{before}\;\mathrm{treatment}–\mathrm{Total}\;\mathrm{score}\;\mathrm{after}\;\mathrm{treatment}\right)\\ \;\times 100/\mathrm{Total}\;\mathrm{score}\;\mathrm{before}\;\mathrm{treatment} \end{array}$$

where the total score before and after treatment is based on the measures above.

The standard comprehensive effect:

•clinical cure: curative effect ≥ 90%

•obvious effects: 75% ≤ curative effect < 90%

•effective: 35% ≤ curative effect < 75%

•invalid: curative effect < 35%

The efficiency of a treatment is calculated as:

$$\begin{array}{l} \mathrm{Efficiency}\;\left(\%\right)=\left(\mathrm{Number}\;\mathrm{of}\;\mathrm{clinical}\;\mathrm{cures}\right)\\ \;+\mathrm{Number}\;\mathrm{of}\;\mathrm{cases}\;\mathrm{with}\;\mathrm{obvious}\;\mathrm{effects}\\ \;\left(+\mathrm{Number}\;\mathrm{of}\;\mathrm{effective}\;\mathrm{cures}\right)\\ \;\times 100/\mathrm{Total}\;\mathrm{number}\;\mathrm{of}\;\mathrm{cases} \end{array}$$

### Secondary outcomes

The average daily consumption of non-steroidal, anti-inflammatory drugs.

ADL performance will be assessed using the modified Barthel index (MBI) [36], which is based on 10 items and is scored on a scale from 0 to 100. A higher score indicates more independence [37].

QOL will be evaluated using the medical outcomes short form 36 health questionnaire (SF-36). The SF-36 has been found to be reliable and is easy to administer [38-40]. The questionnaire has eight scales for different domains of health as follows: physical functioning, bodily pain, physical state, general health, vitality, emotional state, social functioning and mental health. Each scale is scored between 0 and 100 with 100 representing the best score possible. The PCS (physical component scales, summary scales of physical functioning, bodily pain, role-physical and general health) in SF-36 will be the main focus of this investigation as it is a composite of all scales representing the physical aspects of health.

### Safety assessments

Trial coordinators and therapists at each CHC will record adverse events and the details of these events, throughout the study and during the follow-up period. Participants will be able to report adverse events at any time during the trial and will receive advice accordingly. Serious adverse events will be reported to the Human Research Ethics Committee (HREC) of FJTCM.

### Economic evaluation

The cost of interventions will be assessed during the rehabilitation period, including the length of any hospital stay, emergency department visits, general medical treatment, visits to a specialized physician and nursing care. The intervention costs of high-grade hospitals will be compared to those of CHCs. Medicare national fee structures will be used to evaluate the costs of these services since out-of-pocket expenses for these services often do not accurately reflect the service value. Finally, the number of workdays lost by participants during rehabilitation will be used to assess indirect costs.

### Statistical analysis

Statistical analysis will be conducted using SAS software (version 8.2, SAS Institute, Cary, NC, USA). All tests will be two-tailed with *P* values of less than 0.05 considered statistically significant.

Descriptive statistics will be presented for each group as the mean ± standard deviation and all enumeration data will be presented as constituent ratio and rate in the outcomes from baseline to each time point. Changes in differences between groups in the primary and secondary outcomes, before and after treatment and during the follow-up period (at 4 weeks and 12 weeks), will be analyzed using repeated measures ANOVA or a rank-sum test.

Changes in effect sizes between pre- and post-intervention will be performed using the Wilcoxon test and chi square test. The effect size will be evaluated with safety set (SS), full analysis set (FAS), per-protocol set (PPS) and cost-effectiveness analysis. Demographic and CHC characteristics, as well as baseline data, will be analyzed using the FAS to assess the baseline and CHC comparability between groups. The primary analysis of the data will be undertaken using FAS with the principle of intention-to-treat and the PPS. The secondary analysis of the data will be undertaken using PPS. To account for missing data, multiple imputation of missing follow-up measures will be performed per the methods used for missing data [41]. The PPS will be used to assess adherence, the SS to assess adverse events and the cost-effectiveness analysis to assess health economics.

## Discussion

KOA is a major public health problem and this project, funded by the State Administration of Traditional Chinese Medicine, has two aims. First, the curative effect of CTCMRP for KOA rehabilitation will be assessed and CTCMRP will be examined to develop a SRPTCM for KOA rehabilitation in China and worldwide. Secondly, self-management for KOA patients will be improved and their financial burden reduced.

In this study, the rehabilitation therapists will undergo rigorous training and accreditation to administer the CTCMRP intervention. Regular meetings will be held throughout the study to exchange views. The outcome measures will include self-reported measures of pain, the SF-36, QOL and activities of daily living. Knee ROM, the 6 MW test, the stair-climbing test and the comprehensive effect will assess functional performance, strength and physical activity levels. In addition, a health economic assessment will be used to justify the cost-effectiveness of treatments in the current economic climate and a safety assessment will be done to assess adverse events.

The biggest limitation of this protocol is that there is no placebo control and it is not double-blinded. However, the evidence-based medicine center will be blind to the intervention to decrease possible bias. The study also lacks long-term follow-up and evaluation. The 4-week intervention period reflects what is done in actual clinical practice and is long enough to test whether a rehabilitation modality is effective in the short term and whether the efficacy is sustained during the follow-up period. Due to ethical considerations, non-steroidal anti-inflammatory drugs will be permitted as required.

If successful, this project offers a low-cost solution for efficiently linking millions of hospitalized KOA patients with effective outpatient treatment. CTCMRP rehabilitation therapy for KOA can be done in a CHC or at home instead of in a high-grade hospital, thus reducing the financial burden for the patient.

## Trial status

Recruitment is ongoing.

## Abbreviations

6 MW: Six-minute walking test; ADL: Activities of daily living; CHC: Community health center; CT: Conventional therapy; CTCMRP: Comprehensive traditional Chinese medicine rehabilitation protocol; FAS: Full analysis set; FJTCM: Fujian University of Traditional Chinese Medicine; KOA: Knee osteoarthritis; MBI: Modified Barthel index; MMT: Manual muscle test; MRC: Medical Research Council; PPS: Per-protocol set; QOL: Quality of life; ROM: Range of motion; SCT: Stair-climbing test; SF-36: Medical outcomes short form 36 health questionnaire; SRPTCM: Standard rehabilitation protocol in TCM; SS: Safety set; TCM: Traditional Chinese medicine; TDP: *Te Ding Dian Ci Pu*; VAS: Visual analogue scale.

## Competing interests

The authors declared that they have no competing interests.

## Authors’ contributions

HY participated in the design of the study and drafted the manuscript. YS and LC conceived of the study, designed it and participated in reviewing the manuscript. GZ provided guidance on study design, measures and analysis. XL conducted pilot studies to establish the feasibility of the trial. BC helped to conceive the study and participated in reviewing the manuscript. BZ participated in the design of the study and designed study analyses and measures. QZ revised the study protocols and participated in reviewing the manuscript. All authors read and approved the final manuscript.

## Authors’ information

Hu Yan is a doctoral student at Fujian University of Traditional Chinese Medicine, majoring in orthopedics and traumatology in traditional Chinese medicine. Professor Youxin Su MD is a doctoral supervisor at Fujian University of Traditional Chinese Medicine, Department of Research, in the faculty for the Rehabilitation of OA. Professor Lidian Chen MD is the president of Fujian University of Traditional Chinese Medicine, in the faculty for Rehabilitation using Traditional Chinese Medicine. Professor Guohua Zheng MD works at Fujian University of Traditional Chinese Medicine, Institute of Integrated Traditional Chinese and Western Medicine, in the faculty of Statistics. Xueyi Lin, Baojun Chen, Qing Zhang and Bihong Zhou are postgraduate students at Fujian University of Traditional Chinese Medicine, majoring in orthopedics and traumatology in traditional Chinese medicine.

Youxin Su is joint first author.
